# Research on Centrifugal Pump Speed Measurement Based on Vibration Measurement

**DOI:** 10.3390/s25103095

**Published:** 2025-05-14

**Authors:** Yin Luo, Hang Yan

**Affiliations:** National Research Center of Pumps, Jiangsu University, Zhenjiang 212013, China; 2222211021@stmail.ujs.edu.cn

**Keywords:** centrifugal pump, dominant frequency, rotational speed measurement, Zoom-FFT, non-invasive measurement, experimental validation

## Abstract

Traditional rotational speed measurement methods, such as invasive sensors and visual recognition technologies, are often constrained by physical wear and environmental limitations. This paper introduces a non-invasive rotational speed measurement approach based on vibration signal frequency spectrum analysis. The proposed method utilizes the Zoom-FFT algorithm to process vibration signals collected during pump operation, enabling the precise identification of the dominant frequency and its correlation with the pump shaft frequency for accurate speed calculation. The experimental results obtained from a centrifugal pump under varying operating conditions demonstrate the following: At a constant rotational speed, flow variations have a minimal impact on the measurement accuracy, with errors ≤0.04%. Under constant flow conditions, the speed calculation accuracy achieves an error rate of 0.27% across different speeds. Compared to traditional methods, the proposed approach exhibits superior reliability and accuracy. This non-invasive method minimizes physical wear and reduces dependency on environmental factors, offering an effective solution for mechanical equipment monitoring and fault diagnosis.

## 1. Introduction

In modern industry and research, rotational speed measurement is a fundamental and critical technology, playing a vital role in ensuring the efficient and stable operation of machinery. Rotational speed, defined as the number of revolutions a rotating body makes around an axis per unit of time, is not only a basic parameter for assessing the performance of rotating machinery but also a crucial indicator for monitoring and controlling the state of mechanical systems [1]. From wind turbines and pumps to household appliances and precision instruments, accurate rotational speed measurement is indispensable for optimizing performance, preventing failures, and extending the lifespan of these devices. It is a foundational technology in mechanical engineering and manufacturing, as well as a key method for innovation and maintaining operational stability across various high-tech fields [2]. With technological advancements, there is an increasing demand for accuracy, convenience, and non-intrusiveness in rotational speed measurement methods, driving continuous progress and innovation in related technologies.

Centrifugal pumps, as essential tools for energy conversion and fluid transfer, have played an indispensable role in China’s economic development at various stages due to their efficient, stable, and reliable operational characteristics [3]. Rotational speed can comprehensively reflect the complex conditions during pump operation, making it a crucial tool for evaluating pump performance. Although traditional speed measurement techniques are widely used across industries, the continuous advancement of industrial automation and smart manufacturing technologies presents growing limitations and challenges to these methods.

At present, both in China and internationally, rotational speed measurement technologies can be broadly categorized into two main types: analog measurement techniques and digital measurement techniques [4]. Analog methods, such as the tachogenerator method, determine the speed and rotational direction of a motor by using a tachogenerator as a generator and detecting the magnitude and polarity of its back electromotive force (EMF). This approach allows for a straightforward derivation of the relationship curve between the rotational speed and the output voltage. However, in high-speed or low-speed environments, the actual output of this method may deviate from the expected characteristics, resulting in significant measurement errors.

Digital measurement methods, on the other hand, determine rotational speed indirectly by analyzing the continuous pulses generated by digital signals. By estimating speed through the frequency or time intervals of the pulse signals, this technique exhibits high accuracy and stability. Furthermore, digital methods demonstrate superior performance in handling complex signals and adapting to various operating conditions, making them the preferred choice in the field of rotational speed measurement [5]. Nevertheless, conventional measurement methods, whether analog or digital, may encounter challenges, such as measurement errors, installation difficulties, and safety concerns, in practical applications. As a result, there is a pressing need for more precise and reliable speed detection techniques in engineering practices.

Both in China and internationally, researchers have conducted extensive investigations into this subject and proposed various innovative measurement techniques. Antoni investigated the issue of machine tool speed estimation based on vibration signals and proposed a two-step estimation method that combines phase demodulation with joint time–frequency analyses [6]. Researchers at the Inner Mongolia University of Science and Technology analyzed the close relationship between the low-frequency components of noise signals and rotational speed, performing power spectrum and correlation analyses on collected acoustic signals to determine the speed [7]. Zhang Shuai introduced a new method for the time-domain analysis of vibration signals, combining the Hilbert envelope and autocorrelation to calculate the engine speed. Yu Fang and his team delved into the theory of discrete spectrum correction and developed a novel engine speed measurement method based on vibration and discrete spectrum correction techniques. Pan Zhongyong analyzed pressure pulsations caused by rotor–stator interactions within centrifugal pumps and applied Fast Fourier Transform (FFT) to the pressure pulsation signals at the pump outlet [8]. He successfully utilized the blade-passing frequency to achieve the accurate speed measurement of the pump. Tian Liyong proposed using acceleration sensors to collect vibration signals and applying the Zoom-FFT transformation to the acquired signals. This method outputs vibration spectrum curves, enabling the fault diagnosis of sewage pumps by comparing the frequency components of the spectrum under normal operation and fault conditions, processed using both standard FFT and Zoom-FFT [9]. Zhang Zhenhua utilized the Zoom-FFT algorithm to enhance the frequency resolution within the bandwidth around the shaft frequency, achieving refined spectral processing and successfully extracting the shaft count of the propeller for underwater acoustic targets [10].

Given the limitations of common rotational speed measurement methods, the development of novel measurement techniques has become a critical task for both the scientific and industrial communities. Since there is a certain correlation between the rotational speed of centrifugal pumps and vibration frequencies, speed measurement can be transformed into the analysis of vibration frequencies [11]. Zoom-FFT is an algorithm specifically designed for frequency analysis, representing an enhanced method based on the Fourier Transform. By segmenting the signal multiple times consecutively and applying FFT to each segment, Zoom-FFT achieves more refined frequency analysis.

In this study, based on the principles of vibration frequency measurement and analysis, a frequency-domain analysis of vibration signals was conducted. A non-invasive rotational speed measurement method, derived from the frequency spectrum of vibration signals, is proposed. Experimental results validate the reliability and effectiveness of this approach.

## 2. Materials and Methods

### 2.1. Methods for Measuring Rotational Speed

This study aims to accurately measure the rotational speed of a centrifugal pump by determining the rotational shaft frequency using vibration signal data collected via vibration sensors, combined with the Zoom-FFT algorithm [12].

In rotating machinery systems, such as centrifugal pumps, the periodic vibrations induced by the driving force of the motor shaft manifest as the shaft frequency and its harmonic components. By using an accelerometer to collect vibration signals from the casing, the signals are processed through Fourier Transform to generate a frequency spectrum. In the spectrum, the height of each spectral line represents the vibration amplitude of the harmonic at the corresponding frequency. During the normal operation of the unit, vibration frequencies primarily appear as components related to the mechanical rotational speed. Therefore, rotational speed can be determined in real time through spectral analyses and simple calculations.

The primary frequency refers to the dominant vibration frequency generated by the periodic excitation of the mechanical system. It is typically directly related to the shaft frequency or its integer multiples and appears in the vibration spectrum as sharp peaks with high energy, often accompanied by distinct harmonic clusters. In this study, the extraction of the primary frequency, $f_{0}$, follows multiple constraints. First, a threshold is applied to filter candidate peaks with energy levels higher than the average spectral line. Subsequently, the candidate peaks must satisfy the relationship between adjacent frequency points:

(1) $$S\left(f_{n-1}\right)<S\left(f_{n}\right)<S\left(f_{n+1}\right)$$

where $S\left(f_{n}\right)$ denotes the spectral energy density of the signal at frequency $f_{n}$.

Based on the frequency information extracted from the fundamental frequency, the pump speed, $N_{0}$, can be calculated using the following formula:

(2) $$N_{0}=\frac{f_{0}*60}{a}$$

where $N_{0}$ is the pump speed, $f_{0}$ is the fundamental frequency, and a is a positive integer. In many mechanical systems, the fundamental frequency is often an integer multiple of the shaft frequency. Equation (2) establishes the relationship between the fundamental frequency and the shaft frequency, enabling the conversion of the extracted frequency results into the pump speed. An example calculation: the vibration acceleration spectrum indicates a primary frequency of 130.6 Hz. Using the aforementioned method to calculate rotational speed, the speed is determined as

$$N_{0}=\frac{130.6*60}{3}=2612$$

Thus, the current rotational speed is calculated to be 2612 rpm.

### 2.2. Wavelet Denoising

In vibration signal analysis, denoising is a critical preprocessing step for extracting meaningful features [13]. Mechanical vibration signals are often accompanied by environmental noise and measurement interference, and traditional frequency-domain filtering methods may result in the loss of transient impact components. Wavelet transform, with its time-frequency localization properties, enables the adaptive separation of noise and useful signals. By performing multi-scale decomposition to identify the noise frequency bands and applying threshold quantization to detail the coefficients before reconstruction, wavelet denoising can significantly improve the signal-to-noise ratio while preserving singularity information. This provides a high-quality data foundation for subsequent tasks, such as time-frequency analysis and fault diagnosis.

Wavelet denoising is primarily based on the unique characteristics of wavelet transform. Its fundamental principle involves removing noise from a signal using wavelet techniques and recombining the signal to extract useful information [14]. The basic steps of wavelet packet denoising are as follows:

(1) Perform wavelet packet decomposition on the collected vibration acceleration signal.

(2) Select an appropriate wavelet packet basis function using the minimum cost principle.

(3) Determine a suitable threshold based on the noise level of the signal and the characteristics of the target signal.

(4) Set the low-amplitude wavelet coefficients to zero to suppress noise effectively.

(5) Reconstruct the signal using wavelet packet reconstruction, resulting in the target signal after denoising.

### 2.3. Fourier Transform

The definition of the Fourier Transform (FT) can be derived from the concept of the Fourier series, as expressed in Equation (3).

(3) $$X\left(f\right)=\int_{-\infty }^{+\infty }x\left(t\right)e^{-j2\pi ft}dt$$

FT is a widely recognized tool in the field of frequency analysis, offering a powerful means to convert time-domain signals into their equivalent representations in the frequency domain. However, traditional Fourier Transform methods have several notable limitations.

First, the computational process of Fourier Transform is highly complex. Even when discretized and processed using high-speed computers, it remains time-consuming and cannot meet the requirements for rapid detection. Second, traditional FT has inherent limitations when analyzing broadband signals or when a detailed analysis of specific narrowband components within a broadband signal is required. In such cases, the resolution of FT may be insufficient to accurately distinguish the frequency components in the target region [15].

To address this limitation, this paper proposes the use of an improved spectral refinement algorithm. There are various methods for refining a spectral analysis, with common algorithms including the Fast Fourier Transform based on Fourier Series (FFT-FS), the Chirp Z-Transform (CZT), and the Zoom-Fast Fourier Transform (Zoom-FFT). Both the FFT-FS and the CZT algorithms share a similar principle: without increasing the data length, they utilize different mathematical approaches to interpolate and increase the number of FFT points within a selected frequency band [16]. This improves the computational resolution of the signal and enables localized spectral refinement. However, these methods do not enhance the physical resolution and, therefore, cannot improve the frequency discrimination capability of the signal. Additionally, they are limited to refining the spectrum within a specific frequency band.

The Zoom-FFT algorithm, on the other hand, allows for the refinement of spectral structures within any narrowband on the frequency axis. It achieves more detailed spectral results with the same number of transformation points. Furthermore, at the same frequency resolution, the Zoom-FFT algorithm requires fewer Fourier transformation points compared to the FFT-FS and the CZT algorithms. As a result, Zoom-FFT is particularly suitable for multi-frequency analyses, resolving closely spaced frequencies, and applications where fewer transformation points are desired.

**Figure 1:**
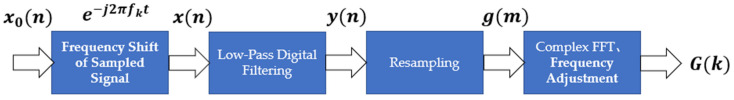
Workflow of the frequency-shifting Zoom-FFT algorithm.

The Zoom-FFT algorithm operates through a series of steps: frequency shifting, anti-aliasing filtering, resampling, complex FFT processing, and frequency adjustment. This process effectively zooms into the desired frequency band, offering a detailed view of the frequency components within that band [17]. By concentrating computational resources on the specified band, the algorithm optimizes processing efficiency and enhances the accuracy of frequency analysis. Currently, the most commonly implemented method for Zoom-FFT is the frequency-shifting approach [18], and its algorithmic workflow is illustrated in Figure 1.

The implementation steps of the Zoom-FFT algorithm are as follows:

(1) Sampling setup: according to the sampling theorem, to prevent aliasing in the frequency domain, the sampling frequency is set as $f_{s}$, and the number of FFT points is N [19].

(2) Frequency shifting: The sampled signal is frequency-shifted to move the frequency band of interest to a lower frequency range, typically near zero frequency. Let $x_{0}\left(n\right)$ represent the original time-domain signal, which is multiplied by $e^{-j2\pi f_{k}t}$, as shown in Equation (4). This operation effectively shifts the frequency components near $f_{k}$ to the origin, forming a new signal $x\left(n\right)$.

(4) $$x\left(n\right)=x_{0}\left(n\right)\cdot e^{-j2\pi f_{k}t}$$

(3) Anti-aliasing filtering: After frequency shifting, the frequency-shifted signal must undergo anti-aliasing filtering to prevent aliasing during the resampling process. Let the impulse response of the low-pass filter be $h\left(t\right)$, and the filtered signal can be expressed as

(5) $$y\left(n\right)=x\left(n\right)\cdot h\left(t\right)$$

(4) Resampling: The filtered signal is then resampled to reduce the computational burden of the subsequent FFT. The resampling frequency is set to $f_{s}/D$, and the resulting signal is denoted as $g\left(m\right)$.

(5) FFT calculation: perform an FFT computation of length N on the resampled data.

(6) Frequency axis adjustment: the frequency axis is adjusted from the resampling frequency $f_{s}/D$ back to the original sampling frequency $f_{s}$, with the initial frequency shift $f_{k}$ added to it.

(6) $$G_{k}=\frac{k*f_{r}}{N}+f_{k}$$

Here, k represents the frequency index. By following these steps, the Zoom-FFT algorithm provides higher frequency resolution within a specific frequency band [20], making it suitable for applications requiring detailed spectral analyses. The following pseudocode (Algorithm 1) illustrates the process of the vibration signal data analysis conducted in this study:
**Algorithm 1:** Zoom-FFT with Wavelet DenoisingInput: Vibration signal data, [f_low, f_high], noise_level = 0.05, zoom_factor = 10Output: Enhanced Zoom-FFT spectrumProcedure:1. Signal Acquisition:  - Read time(t) and vibration(z_data) from file  - Compute fs = 1/Δt2. Preprocessing:  - Add noise: z_noisy ← z_data + N(0, noise_level2)  - Wavelet denoising (db4, level = 5):   * Estimate threshold via median absolute deviation   * Apply soft-thresholding (α = 2)   * Reconstruct z_denoised3. Zoom-FFT Core:  a. Frequency shift to baseband:   z_shifted←z_denoised · e^{−j2πf_center t}   where f_center = (f_high + f_low)/2  b. FIR lowpass filtering (order = 100, cutoff = bandwidth/2)  c. Downsample by decimation_factor = max (1, ⌊fs/(2·bandwidth·zoom_factor)⌋)  d. Apply Hamming window and compute FFT  e. Adjust frequency axis: f_zoom = f_center + (k·fs_new/N − fs_new/2)4. Comparison:  - Compute conventional FFT with same window  - Plot both spectra over [f_low, f_high] with matched axes (0–0.008)Critical Parameters:- Bandwidth = f_high - f_low- fs_new = fs/decimation_factor- Threshold: σ = median(|cD1|)/0.6745

## 3. Experimental Setup and Method

### 3.1. Experimental Setup

**Figure 2:**
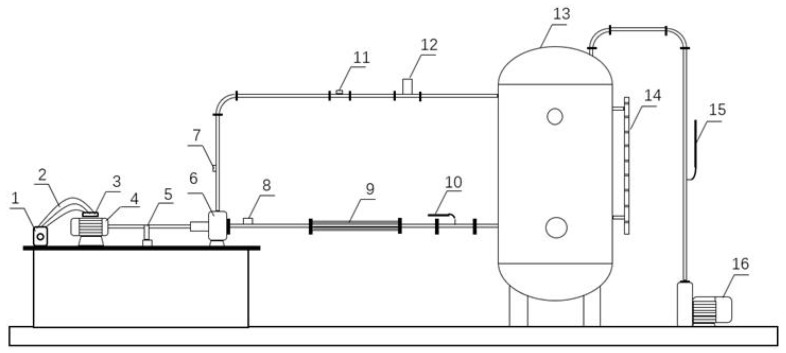
Centrifugal pump test bench. 1. Frequency converter. 2. Electric cable. 3. Motor junction box. 4. Centrifugal pump drive motor. 5. Coupling. 6. Centrifugal pump. 7. Outlet pressure sensor. 8. Inlet pressure sensor. 9. Bellows. 10. Inlet ball valve. 11. Flowmeter. 12. Pipeline outlet valve. 13. Water storage tank. 14. Magnetic level indicator. 15. Vent valve. 16. Vacuum pump.

To validate the correlation between the vibration characteristics and the rotational speed of the centrifugal pump, experimental studies were conducted on a centrifugal pump system. The experimental setup was designed as a closed-loop system, as illustrated in Figure 2.

**Figure 3:**
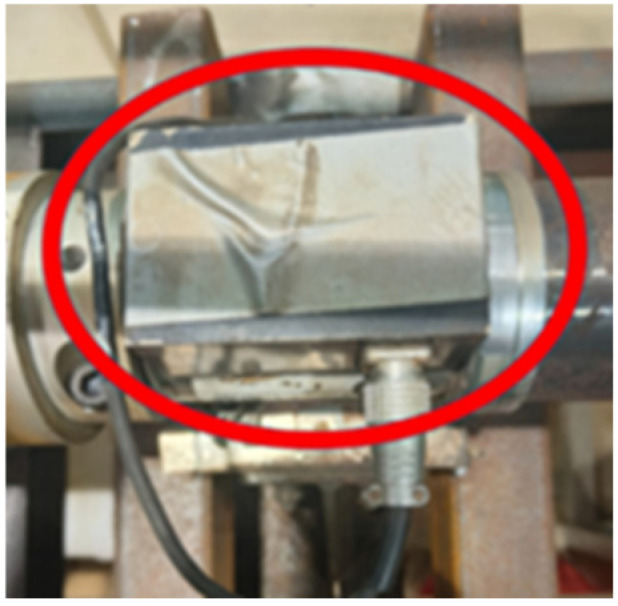
Torque transducer.

**Figure 4:**
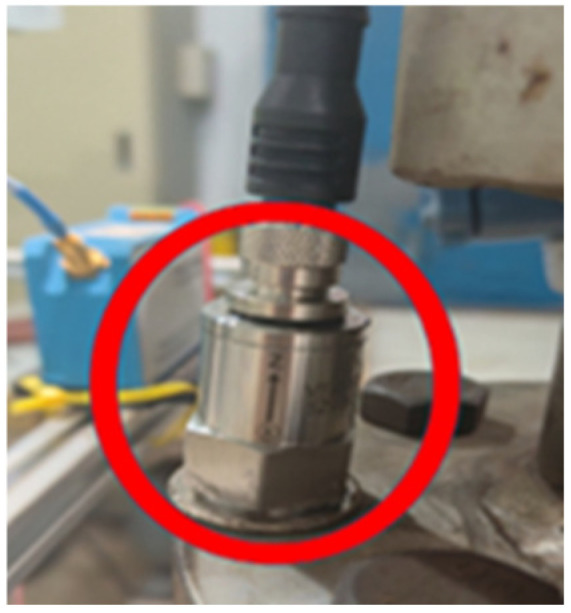
Vibration sensor.

The main components of the experimental system include a single-stage, single-suction model centrifugal pump (parameters listed in Table 1); a water tank; stainless steel piping; a motor (parameters listed in Table 2); pump pipeline control valves; a torque meter (Figure 3); a high-precision electromagnetic flowmeter; and a VTall-S101L-2 temperature and vibration sensor (Figure 4).

### 3.2. Experimental Method

The rated speed of the pump used in this experiment is 2950 r/min. To enhance the reliability of the analysis results, the rotational speed was adjusted using a frequency converter, and vibration signal data were collected at pump speeds near 2400 r/min, 2600 r/min, and 2800 r/min. During the operation of the centrifugal pump, performance parameters were collected by sensors and transmitted to a computer program via a data acquisition board. The data acquisition program was developed using LabVIEW [21].

**Figure 5:**
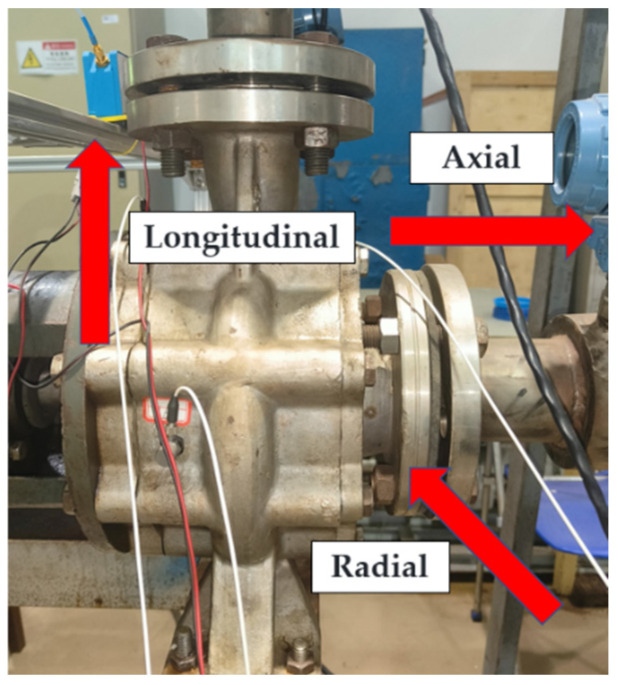
Vibration signal monitoring points.

During the experiment, the rotational speed, torque, and other parameters of the pump system were monitored and displayed using a combination of a torque meter and a display unit. The experiment employed an accelerometer calibrated in accordance with the ISO 16063-21 standard, with its calibration frequency range extending to three times the vibration frequency of the centrifugal pump [22]. The sensor was securely mounted using a magnetic base, ensuring a vertical alignment deviation of less than 3 degrees, relative to the pump surface. A sampling frequency of 1000 Hz was selected, equivalent to 20 times the frequency corresponding to the pump’s maximum rotational speed. Throughout the experiment, the environmental conditions, including temperature (maintained at 20 ± 2 °C) and humidity (50 ± 10%), were continuously monitored to minimize the impact of environmental fluctuations on the sensor performance. Data acquisition was conducted during the stable operation of the pump unit, with multiple datasets collected for subsequent analysis. Signal monitoring points were determined based on the vibration measurement and evaluation standards outlined in ISO 7919 (GB/T 11348) [23]. The specific vibration signal monitoring locations used in this study are shown in Figure 5.

## 4. Results and Discussion

### 4.1. Data Processing

The vibration data collected at the test points were processed using the Zoom-FFT algorithm, resulting in frequency spectra corresponding to different flow rates and rotational speed conditions.

**Figure 6:**
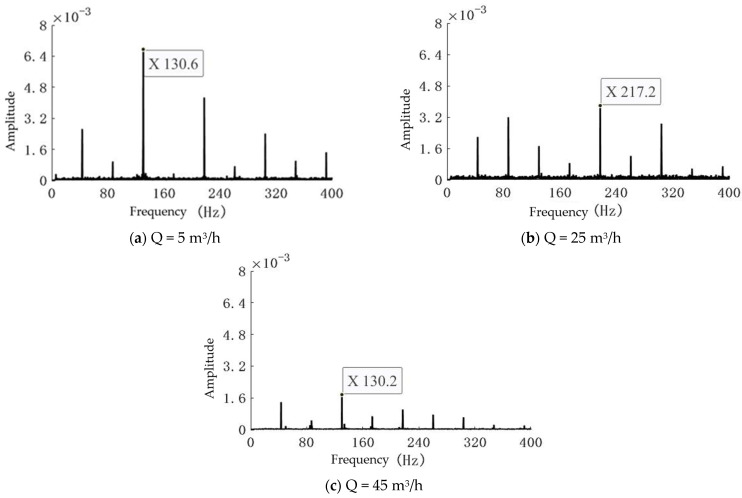
Frequency spectra of vibration signals at different flow rates.

By processing the experimental data, three frequency spectra corresponding to flow rates of 5 m^3^/h, 25 m^3^/h, and 45 m^3^/h at a centrifugal pump speed of 2600 r/min were obtained, as shown in Figure 6.

**Figure 7:**
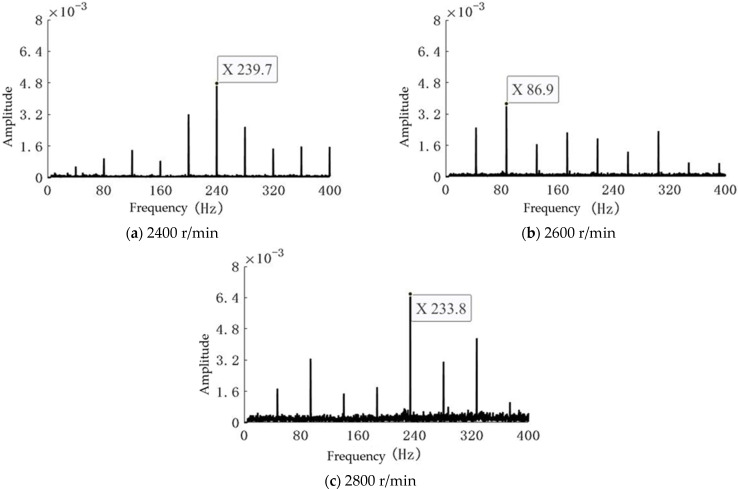
Frequency spectra of vibration signals at different rotational speeds.

The Zoom-FFT algorithm was applied to process the acquired vibration signals, generating frequency spectra for the centrifugal pump operating under a constant flow rate of 20 m^3^/h. As depicted in Figure 7, the spectral results correspond to three distinct rotational speeds: 2400 r/min, 2600 r/min, and 2800 r/min.

In this study, the refined Fourier Transform (Zoom-FFT) was employed to process the vibration signals of the centrifugal pump, enabling the determination of the pump’s shaft frequency. The fundamental frequency, as a multiple of the shaft frequency, serves as a critical indicator of the pump’s rotational speed. Based on the fundamental frequency, the rotational speed of the centrifugal pump was further calculated using the rotational speed formula (Equation (2)).

Experimental data under varying rotational speeds, but at the same flow rate, were processed and analyzed using Equation (2). By comparing the vibration signal spectra across different operating conditions, a special emphasis was placed on the characteristics of the fundamental frequency to verify the applicability of the rotational speed formula.

As shown in Table 3, the influence of varying flow rates on the rotational speed measurements under the same rotational speed was minimal, with negligible errors.

Under the condition of varying rotational speeds at the same flow rate, as presented in Table 4, the calculated rotational speeds closely matched the actual motor speeds. This indicates that at the same rotational speed, the fundamental frequency characteristics extracted from the vibration signal spectrum exhibit a high degree of consistency with the actual speed. This demonstrates the accuracy and reliability of the proposed measurement method under different flow rate conditions.

During the calculation of a centrifugal pump’s rotational speed, there may be instances where accurate speed values cannot be obtained. Such discrepancies are typically not caused by inaccuracies in the speed calculation method itself, but rather by factors such as the vibration characteristics of other structural components inside the pump or the improper selection of monitoring points. These issues can interfere with the extraction of the fundamental frequency during a spectrum analysis, thereby affecting the results.

Specifically, this issue is more likely to occur when the fundamental frequency in the vibration signal is an integer multiple of the shaft frequency. The shaft frequency refers to the vibration frequency generated by one complete revolution of the pump shaft. During actual operation, in addition to the blade-passing frequency, other structural components of the pump—such as bearings, seals, and wear rings—also produce specific vibration frequencies. These frequencies often appear as integer multiples of the fundamental frequency in the spectrum analysis, especially when the monitoring point is located near these structural components, where their influence becomes more pronounced.

Moreover, the location of the monitoring point is a critical factor affecting the results of the spectrum analysis. Monitoring points at different locations may capture vibration signals of varying intensities, which can influence the extraction of the fundamental frequency. If the monitoring point is positioned near certain specific structures of the centrifugal pump, the vibration signals generated by these structures may dominate the spectrum analysis, potentially masking the vibration characteristics caused by the blade-passing frequency.

When calculating the rotational speed of a centrifugal pump, it is essential to comprehensively consider the vibration characteristics of various structural components within the pump and the selection of the monitoring point location. This ensures the accurate extraction of the fundamental frequency that reflects the pump’s rotational speed. In practical applications, it may be necessary to adjust the monitoring point location or employ more advanced signal processing techniques to distinguish between vibration signals from different sources. This improves the accuracy and reliability of rotational speed calculations.

This challenge highlights the importance of accounting for the complexity of the system structure and the strategic selection of monitoring points in a mechanical vibration analysis. By gaining a deeper understanding of the vibration characteristics of the pump’s internal structures and optimizing the layout of monitoring points, the accuracy of rotational speed measurements can be significantly enhanced. This provides more reliable data support for the performance monitoring and fault diagnosis of centrifugal pumps.

### 4.2. Error Validation

To verify the reliability and accuracy of the fundamental frequency extraction method and the rotational speed calculation formula, multiple experiments were conducted. The results, as shown in Table 4, indicate that the proposed method achieves a rotational speed calculation accuracy of 0.27%. The rotational speeds obtained through the fundamental frequency extraction and calculation formula exhibit a high degree of consistency with the actual operating speeds of the pump, meeting the requirements for rotational speed measurement.

During the experiments, vibration signal data were collected across operating conditions ranging from 5% to 100% of the rated rotational speed, and the proposed method was employed for speed calculation. The primary source of errors in the low-speed range (5–30% of the rated speed) was the weak signal, which resulted in a low signal-to-noise ratio (SNR). Consequently, the spectral main peak was obscured by background noise, leading to the inaccurate identification of the fundamental frequency. In the medium-to-high speed range (30–100% of the rated speed), errors were primarily caused by harmonic interference and mechanical resonance. Harmonic amplitudes of multiples of the rotational frequency exceeded the fundamental frequency, or resonance peaks unrelated to the rotational speed appeared, causing sensor output saturation. Despite these error factors, the overall error was maintained within an acceptable range, further demonstrating the reliability of the proposed method. During the experiments, factors such as noise interference during signal acquisition, the resolution of the Zoom-FFT algorithm, and the number of blades were identified as potential sources of errors. Despite these sources of error, the overall error was controlled within an acceptable range, further confirming the reliability of the proposed method.

## 5. Conclusions

This study proposed a novel method for measuring the rotational speed of centrifugal pumps based on the extraction of the dominant frequency from vibration signals. The theoretical foundation and implementation of this approach were validated through an experimental analysis.

1. This research systematically compared the Fourier Transform (FT) with the refined Zoom-Fast Fourier Transform (Zoom-FFT) and provided a detailed explanation of the principles underlying Zoom-FFT. The comparative analysis highlighted the significant advantages of Zoom-FFT in handling high-frequency details and enhancing the spectral resolution. These findings establish a solid theoretical basis for a subsequent vibration signal analysis.
2. By analyzing the vibration characteristics of centrifugal pumps, it was observed that the dominant frequency is an integer multiple of the pump shaft rotation frequency, directly reflecting the pump’s rotational speed. Based on this observation, a rotational speed calculation formula was proposed, enabling the precise determination of the actual speed of the centrifugal pump.
3. The experimental data analysis compared the results of the dominant frequency extraction and the rotational speed calculation with the actual operating conditions of the pump equipment. As demonstrated by the data in Table 4, the computational results show excellent agreement with the actual pump operating speeds, achieving a remarkable calculation accuracy of 0.27%, which exceeds the precision standards of conventional measurement methods.

In summary, the proposed vibration-signal-based method for measuring the rotational speed of centrifugal pumps achieves accurate speed determination through dominant frequency extraction and rotational speed calculation. This approach provides a crucial basis for the performance monitoring and condition assessment of pump equipment. The findings of this study advance the application of vibration signal analyses in engineering and offer new technical support and methodologies for the monitoring and maintenance of centrifugal pump operation.

## Figures and Tables

**Table 1:** Geometric parameters of test pump.

Parameter	Symbol and Unit	Value
Design flow rate	Q/m^3^/h	50
Design head	H/m	34
Rotational speed	r/min	2950
Specific speed	$n_{s}$	81.5
Number of blades	Z	6

**Table 2:** Main motor parameters.

Parameter	Symbol and Unit	Value
Rated voltage	U/V	380
Rated rotational speed	n/r/min	2950
Rated efficiency	η/%	89.4
Rated power	P/kW	15

**Table 3:** Rotational speed errors at different flow rates under the same rotational speed.

Flow Rate (m^3^/h)	Calculated Rotational Speed (r/min)	Error (%)
5	2612	0.04
25	2606	0.02
45	2604	0.01

**Table 4:** Rotational speed errors at different rotational speeds under the same flow rate.

Actual Rotational Speed (r/min)	Calculated Rotational Speed (r/min)	Error (%)
2400	2367	0.11
2600	2607	0.27
2800	2805	0.20

## Data Availability

The data that support the findings of this study are available from the corresponding author upon reasonable request.
